# Prescription Drug Promotion by Social Media Influencers: A Systematic Scoping Review

**DOI:** 10.1001/jamanetworkopen.2026.2738

**Published:** 2026-03-23

**Authors:** Sascha Gell, Sneha Dave, Erin Willis, Elaina J. Vitale, Steven Woloshin, Raffael Heiss

**Affiliations:** 1 Center for Social and Health Innovation, MCI Management Center Innsbruck, Innsbruck, Austria; 2 Department of Communication, University of Vienna, Vienna, Austria; 3 Lisa Schwartz Foundation for Truth in Medicine, Norwich, Vermont; 4 Generation Patient, Indianapolis, Indiana; 5 Department of Advertising, Public Relations and Media Design, University of Colorado Boulder, Boulder; 6 Dartmouth Biomedical & Health Sciences Libraries, Geisel School of Medicine, Dartmouth College, Hanover, New Hampshire; 7 The Dartmouth Institute for Health Policy and Clinical Practice, Dartmouth College, Hanover, New Hampshire

## Abstract

**Importance:**

Prescription drug promotion by social media influencers (hereinafter *influencers*) is a growing phenomenon that raises concerns about misleading advice, insufficient regulatory oversight, and ethically problematic marketing practices. Understanding the scope and risks of influencers’ promotional activities is essential to protect public health.

**Objective:**

To systematically review existing research on influencer prescription drug promotion, with a focus on identifying recurring themes, regulatory gaps, and opportunities for intervention.

**Evidence Review:**

This systematic scoping review was conducted in accordance with the Preferred Reporting Items for Systematic Reviews and Meta-Analyses reporting guideline. The search strategy was developed with a health sciences librarian and preregistered on Open Science Framework. English-language articles from peer-reviewed journals were retrieved from the Medline, Communication & Mass Media Complete, CINAHL Complete, PsycInfo, Web of Science, Business Source Complete, Scopus, and Cochrane Library databases, covering research conducted in diverse online and health care contexts worldwide from 2004 to 2024. Searches used key terms related to influencers, social media, and prescription drugs, completed on March 13, 2024. Included studies underwent thematic analysis. The primary outcomes included concerns related to accuracy (misinformation), transparency (disclosure practices), and persuasiveness (personal and parasocial narratives) of prescription drug promotion by influencers.

**Findings:**

Twelve articles met inclusion criteria (8 empirical and 4 theoretical), addressing topics such as contraceptive advertising, performance-enhancing drugs, and broader pharmaceutical promotion. Methods included content analyses, interviews, and experiments. Three recurring themes emerged: (1) misinformation stemming from influencers’ limited expertise in the context of audiences’ low health literacy; (2) weak regulatory oversight and inconsistent disclosure practices; and (3) parasocial narratives that blur distinctions between personal testimony and paid promotion.

**Conclusions and Relevance:**

This systematic scoping review study of prescription drug promotion by influencers found that such promotion carried risks of inaccurate or misleading advice, often amplified through personal and emotionally resonant narratives in an environment with limited oversight and enforcement. Despite the small and fragmented evidence base, these findings highlight the urgent need for updated regulatory guidance, standardized and enforceable disclosure requirements, stronger platform accountability, and targeted digital literacy initiatives. Policymakers and researchers should act proactively to safeguard public health.

## Key Points

**Questions:**

What is known about the risks of prescription drug promotion by social media influencers, and how can current evidence inform future research and effective policy?

**Findings:**

In this systematic scoping review of 12 articles published in peer-reviewed journals, social media influencer promotion of prescription drugs was consistently associated with misinformation, weak and outdated regulatory oversight, and audience difficulty in recognizing promotional intent when marketing was embedded in personal narratives.

**Meaning:**

These findings highlight an urgent need for updated regulatory guidance, stronger and standardized disclosure requirements, enhanced platform accountability, and targeted digital literacy initiatives to mitigate public health risks.

## Teaser

This systematic scoping review examines the existing research on social media influencer promotion of prescription drugs and identifies recurring themes, gaps in regulatory oversight, and opportunities for public health intervention.

## Introduction

The prescription drug industry is increasingly partnering with social media influencers (hereinafter *influencers*)—that is, individuals who attract a large number of followers and influence them by sharing engaging content.^1,2^ These influencers are often patients—referred to as *patient influencers*—and post personal stories and experiences, making them persuasive forces.^3,4^ Examples include celebrity influencers such as Khloé Kardashian, Lady Gaga, or gold-medalist Olympic athlete Aly Raisman, who all have promoted migraine medication to millions of followers.^5^ However, beyond celebrity endorsements, smaller influencers also maintain financial relationships with health care companies, for example, by promoting prescription weight loss drugs on social media.^6^

Such collaborations may contribute to the spread of misleading information about prescription drugs on social media, potentially leading to medication misuse and harmful interactions.^3,7^ This may be particularly problematic when such promotions come from health professionals, including physicians.^8^ They also raise public health concerns, as influencer promotions may amplify pharmaceutical demand and encourage inappropriate prescribing or use.^9,10,11^

Influencer-driven prescription drug promotion should be situated within the broader landscape of health misinformation and digital risk communication, where misleading health information is shaped by platform dynamics and persuasive narratives.^12,13^ Despite its growing relevance, influencer-driven prescription drug promotion is not yet well understood. Synthesizing the existing evidence is essential to better understand current practices, assess potential risks, and guide future research and policy. To address this, we conducted a systematic scoping review to examine the current state of such marketing on social media platforms and to identify opportunities for public health intervention.

## Methods

### Study Process

This systematic scoping review followed the Preferred Reporting Items for Systematic Reviews and Meta-Analyses (PRISMA) reporting guideline extension for scoping reviews. The protocol was registered on the Open Science Framework.^14^ A professional health sciences librarian (E.J.V.) collaborated with the research team to develop the search strategy and search string. On March 13, 2024, we searched 8 databases—Medline, Communication & Mass Media Complete, CINAHL Complete, PsycInfo, Web of Science, Business Source Complete, Scopus, and the Cochrane Library—using key terms related to influencers, social media, and prescription drugs. The librarian conducted the search using subject headings and text words adapted to each database’s syntax. Search results were exported to EndNote, and duplicates were removed using a combination of automatic detection, DOI matching, and manual review.

We included English-language journal articles published from 2004 onward (the year Facebook launched in the US), including peer-reviewed empirical studies as well as editorials and commentaries, with a primary focus on influencers, social media platforms, and prescription drugs (eTable 1 in Supplement 1). Influencers were defined as individuals who exert influence through social media platforms, thus we excluded articles using the term *influencer* without reference to social media contexts (eg, offline opinion leaders). After initially removing duplicates, 2549 records remained. Two researchers (S.G. and S.D.) independently screened titles and abstracts; disagreements were resolved through discussion with 3 additional team members (E.W., S.W., and R.H.). After screening was completed, a total of 27 articles underwent full-text review, of which 12 met inclusion criteria (Figure).

**Figure.**
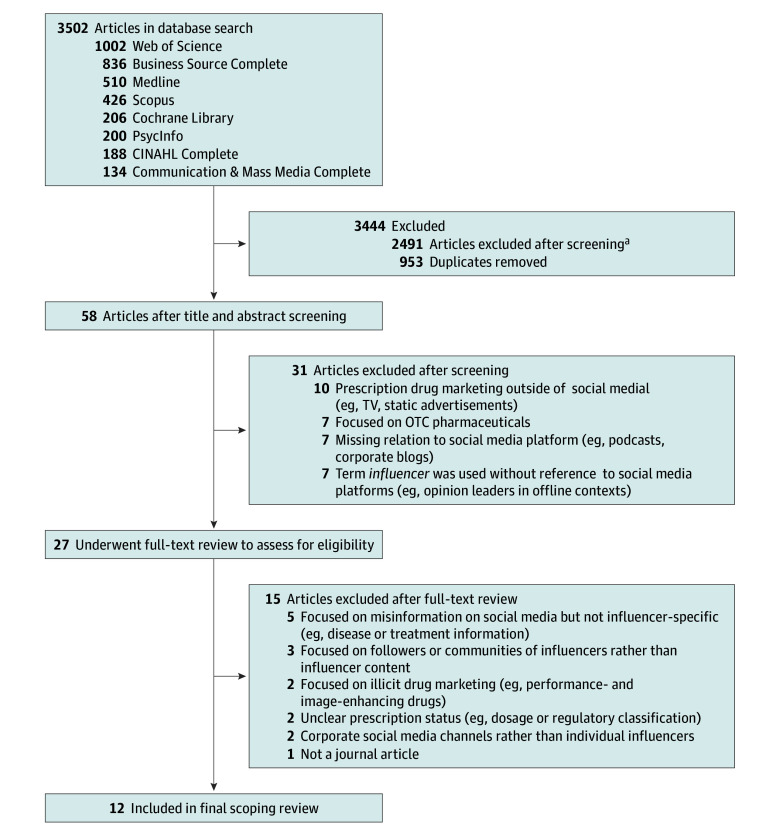
Scoping Review Flowchart OTC indicates over-the-counter. ^a^Individual reasons for exclusion were not counted but included non-English article or abstract, no peer review, not a journal article (eg, news article, book chapter), and lack of focus on main concepts (eg, influencers, social media platforms, prescription drugs).

### Data Analysis

The content of these articles was extracted using a predefined codebook (eTable 2 in Supplement 1) and entered into a shared spreadsheet. The final coding sheet included 7 overarching categories: article metadata, study design, influencer information, influencer content, prescription drug information, main findings, and policy considerations. Thematic analysis was used to descriptively synthesize key findings and map recurring patterns, themes, and gaps across studies.^15^ Two researchers (S.D. and E.W.) independently annotated the material, coded recurring patterns, and met to discuss and refine emerging themes. Discrepancies were resolved through dialogue and discussion. The resulting themes were grouped into 3 overarching categories reflecting concerns related to accuracy (eg, misinformation or omission of evidence-based information), transparency (eg, disclosure of sponsorship or promotional intent), and persuasiveness (eg, the use of personal or parasocial narratives). This approach allowed for a structured understanding of the research landscape, regulatory and policy developments, and potential public health implications of influencer-driven pharmaceutical marketing.

## Results

The 12 included articles^3,9,16,17,18,19,20,21,22,23,24,25^ covered a range of topics, including birth control, performance-enhancing drugs, and general pharmaceutical promotion. They also used diverse methods such as content analyses, interviews, and experiments (Table 1).

**Table 1.** Overview of Included Articles

Source	Study design	Objective	Findings	Themes
Darmawan and Huh,^22^ 2022	Empirical: online experiment	Examine effects of message type and sponsorship disclosure on attitudes and behavioral intentions	Unbranded posts more favorable than branded; effects mediated by persuasion knowledge; disclosure impacted unbranded posts only	Policy, oversight, and ethics; parasocial narratives
Kadakia et al,^16^ 2023	Theoretical: comment	Call for FDA modernization in regulating health misinformation	Recommend simplified labeling; infodemic monitoring; partnerships with tech platforms	Misinformation; policy, oversight, and ethics
Kim,^23^ 2022	Empirical: online experiments	Examine effects of illness disclosure on persuasion knowledge and behavior	Illness narratives reduced persuasion knowledge; increased positive attitudes via transportation and parasocial interaction; enhanced help-seeking	Parasocial narratives
Niburski and Niburski,^24^ 2023	Empirical: observational analysis	Assess effects of Elon Musk’s tweets on search, purchases, and media	Tweets boosted engagement; increased sentiment and purchasing of bupropion hydrochloride (Wellbutrin) and methylphenidate hydrochloride (Ritalin)	Parasocial narratives
Nickles et al,^17^ 2022	Empirical: cross-sectional analysis	Assess content producers, treatments, bias, and referral to care	Podiatrists dominated educational content; bloggers less likely to encourage professional care; financial bias in 15.7%	Misinformation
Paoli and Cox,^18^ 2023	Empirical: content analysis	Analyze legal, ethical, and policy aspects of PIED promotion	26 Influencers promoted PIEDs; often unregulated or illegal markets; focus on performance and appearance; EU laws insufficient	Misinformation; policy, oversight, and ethics; parasocial narratives
Pfender and Devlin,^19^ 2023	Empirical: content analysis	Examine framing of hormonal discontinuation and alternatives	Influencers promoted discontinuation; inaccurate sexual health information	Misinformation
Suzuki et al,^20^ 2024	Empirical: content analysis	Profile physician influencers; fact-check COVID-19 drug tweets	Mostly accurate content; real-name use and fact-checking key to credibility	Misinformation
Thomas,^9^ 2019	Theoretical: feature article	Examine marketing role of paid influencers	Influencers drive sales; ethical concerns over disclosure; shift from reach to engagement	Policy, oversight, and ethics
Trepanowski and Grant-Kels,^25^ 2023	Theoretical: editorial	Highlight non–evidence-based dermatologic advice	Most content from nonexperts; misleading or harmful advice; call for certified dermatologist engagement	Policy, oversight, and ethics
Willis and Delbaere,^3^ 2022	Theoretical: viewpoint	Define industry terms; propose research agenda	Identifies terminology gaps; calls for systematic investigation	Misinformation; policy, oversight, and ethics; parasocial narratives
Willis et al,^21^ 2023	Empirical: qualitative interviews	Explore how patient influencers convey medication information	Emphasized experiential knowledge; staying informed; deferring to physicians	Misinformation; policy, oversight, and ethics

Abbreviations: EU, European Union; FDA, US Food and Drug Administration; PIED, performance- and image-enhancing drug.

As summarized in Table 2, 3 overarching themes characterize the 12 included studies. The themes reflected the main outcome domains. Accuracy was reflected in the theme expressing concern about misinformation; transparency, in questioning the lack of policy, oversight, and ethics related to patient care; and persuasiveness, in understanding sponsored content in the context of parasocial narratives. Parasocial narratives refer to personal storytelling strategies that foster perceived intimacy and 1-sided relationships between influencers and audiences.^12,26^

**Table 2.** Themes in Influencer Promotion of Prescription Drugs

Theme (No. of studies)	Synthesis of study content
Expressing concern about misinformation (n = 7)	Patient-shared experiences widely consumed and may obscure evidence-based information; influencers share content beyond their expertise, and platform features hinder timely correction; algorithms amplify misleading content, and influencers operate across jurisdictions; health content often gain-framed and lacks alternative treatment options; limited health literacy and difficulty assessing health claims
Questioning the lack of policy and oversight, and ethics related to patient care (n = 7)	Regulations do not reflect persuasive strategies and technological change (eg, social features, algorithms); FDA and FTC guidance vague and lacks specificity regarding algorithmic targeting; limited transparency and difficulty identifying sponsored content; regulatory surveillance limited, particularly across borders; calls for stronger partnerships among agencies, industry, and influencers and enhanced monitoring
Understanding sponsored content in the context of parasocial narratives (n = 6)	Personal narratives blur the line between authentic experience and paid promotion; influencers occupy multiple roles (peer, patient, promoter) and increase audience engagement and trust; parasocial dynamics increase persuasiveness and facilitate meaning transfer; platform features (eg, comments, direct messages, ephemeral content) enhance parasocial dynamics

Abbreviations: FDA, US Food and Drug Administration; FTC, Federal Trade Commission.

First, concerns about accuracy and misinformation were raised in 7 studies.^3,16,17,18,19,20,21^ These studies collectively emphasized that health influencers often present content beyond their clinical expertise, exaggerate benefits, and frequently omit alternative treatment options. For instance, one study^19^ found that influencers on a social media platform discussing sexual health commonly shared inaccurate information about contraception and prevention of sexually transmitted infections, despite expressing high confidence in their own knowledge. Another article^21^ showed that patient influencers, while motivated by peer support, often blurred the line between personal experience and promotional messaging, making it difficult for audiences to distinguish authentic testimonials from marketing. However, not all findings were negative. One study analyzing tweets by Japanese physician influencers about COVID-19 treatments^22^ found that most content was accurate, illustrating the potential of clinically trained voices to contribute trustworthy information in social media environments. At the same time, physicians as influencers also bear risks associated with blurred professional and commercial roles, such as when physicians receive industry payments.^9^

Second, existing US Food and Drug Administration (FDA) and Federal Trade Commission (FTC) guidance on transparency was described as vague, outdated, and difficult to enforce across global platforms. Ethical concerns centered on limited transparency, difficulty identifying sponsored content, and insufficient monitoring of algorithmic targeting. Several studies^3,9,16,18,21,22,25^ identified major gaps in the regulation of health-related influencer content. One study^16^ emphasized that current FDA frameworks fail to address unbranded prescription drug promotion on social media, where influencers may omit key safety information and present marketing as personal testimony. Another analysis^9^ illustrated how weak disclosure enforcement and jurisdictional fragmentation limit oversight, citing the FDA’s warning to Kim Kardashian for promoting a morning sickness drug without adequate risk information and noting that UK authorities lack enforcement power over foreign influencers. A third study^21^ found that even well-intentioned patient influencers can obscure pharmaceutical sponsorship within personal narratives, blurring the line between education and promotion. Together, these findings underscore the need for updated, enforceable, and cross-border regulatory frameworks tailored to the evolving dynamics of influencer-driven health communication.

Third, several studies highlighted the critical role of parasocial dynamics in shaping trust and engagement with influencer content (persuasiveness).^3,9,18,22,23,24^ Influencers often occupy overlapping roles as physicians, patients, peers, and promoters, which increases the likelihood that audiences will engage with and trust their medical advice, particularly when framed as emotionally resonant personal stories. For instance, one experimental study^23^ found that illness disclosure narratives in sponsored pharmaceutical posts reduced recognition of persuasive intent and increased favorable attitudes toward the promoted drug, underscoring the persuasive force of parasocial storytelling. Another study^22^ showed that influencers’ personal stories reduced persuasion knowledge, particularly when presented as unbranded disease awareness posts without sponsorship disclosure. Such effects are often linked to the ability of narratives to foster identification with the influencer, but also through aestheticized visuals, emotional vulnerability, and perceived similarity to their audience.^22,23^ Another study^21^ also highlighted that platform features such as direct messaging, comments, and short-form video content can deepen parasocial bonds. A study on Elon Musk’s personal commentary on psychiatric medications^24^ showed that these endorsements triggered measurable increases in online search behavior and treatment interest.

The 3 thematic categories were relatively evenly represented across the included articles. Expressing concern about misinformation^3,16,17,18,19,20,21^ and questioning the lack of policy, oversight, and ethics related to patient care^3,9,16,18,21,22,25^ were each addressed in 7 articles, while “understanding sponsored content in the context of parasocial narratives” was identified in 6 articles,^3,9,18,22,23,24^ indicating that no single theme clearly dominated among the current literature (Table 2).

## Discussion

The reviewed studies raise several concerns that warrant attention. First, there is a clear risk of misinformation, particularly regarding the safety and efficacy of prescription drugs, as influencers often lack medical expertise yet continue to promote these products. For example, Khloé Kardashian’s promotion of the migraine drug rimegepant sulfate included claims that the FDA later deemed false and misleading.^10^ However, even influencers with medical expertise may provide misleading advice when professional authority is entangled with commercial incentives.^8,9^

Second, regulatory oversight remains weak, allowing influencers to operate in largely unmonitored environments with minimal enforcement. Misconduct frequently results only in warning letters, and while prominent influencers may attract scrutiny, smaller influencers often escape detection altogether.^3^ Even in high-profile cases, enforcement is limited: Khloé Kardashian, despite having roughly 300 million followers, received only an untitled FDA letter, a sanction unlikely to deter broader noncompliance.^10^ Although regulatory examples are based on the FDA and FTC, the concerns identified across studies extend beyond the US context. Similar regulatory challenges are found internationally, including fragmented oversight, inconsistent disclosure standards, and limited enforcement capacity for influencer-based health and pharmaceutical marketing in the United Kingdom and European Union.^7^ European efforts such as the Digital Services Act seek to address these gaps through stronger platform governance and coordinated oversight and point toward increasing international regulatory efforts.^12^ Together, these findings suggest that influencer-driven prescription drug promotion represents a cross-national regulatory challenge that requires greater international alignment and enhanced platform accountability.

Third, the impact of inaccurate information spread in a weak regulatory environment may be amplified through personal narratives. Stories of how a drug helped an influencer cope with a condition can be persuasive even in the absence of evidence, consistent with theories of narrative persuasion showing that storytelling increases emotional engagement and identification while reducing critical scrutiny of claims.^27^ This is because social media users often build parasocial bonds with the influencers they follow, or may even engage in real conversations when following microinfluencers, who are more responsive to followers’ comments. This reinforces perceived credibility and builds relational trust. These relational dynamics may be particularly influential in health contexts, where uncertainty, emotional vulnerability, and experiential knowledge play a central role in decision-making.^28^ As a result, many users struggle to distinguish between authentic experience and paid promotion, as illustrated in initial experimental evidence.^23^ Clear and transparent disclosure of the advertising intent is therefore essential to activate people’s persuasion knowledge and support critical evaluation of influencer-promoted prescription drugs.^22^

Collectively, these issues increase the risk that audiences act on misleading or harmful recommendations. Although the evidence base is limited, our findings point to clear directions for future research and regulatory action (Table 3). For instance, more research is needed to examine how real and parasocial ties shape responses to personal stories or drug advice from influencers, to evaluate models for disclosing industry entanglements, and to map enforcement gaps across jurisdictions. Importantly, limited evidence should not justify inaction. Applying the precautionary principle, policymakers can strengthen platform accountability, standardize and enforce sponsorship disclosures, and invest in digital literacy initiatives. Together, stronger evidence and greater political will can form the foundation for addressing this emerging public health challenge.

**Table 3.** Key Issues Linked to Research Needs and Policy Action

Key issue	Concern	Research need	Policy action
Misinformation from unqualified influencers	Inaccurate information on dosage, safety, and off-label use	Study how misinformation spreads; how to flag or contextualize	Incentivize platform moderation; define *misleading* and *harmful*
Parasocial trust reduces critical thinking	Storytelling masks persuasion; mimics peer advice	Study how misinformation spreads; how to flag or contextualize	Mandate visual disclosures; clarify sponsorship
Blurred lines between experience and promotion	Users fail to recognize paid content	Test disclosures; identify vulnerable groups	Standardize disclosure formats; health and advertisement literacy
Pharma-influencer partnerships may bias content	Financial ties skew risk-benefit framing	Analyze pharma incentives and messaging	Require public registers of pharma-sponsored posts and influencers and transparent disclosures.
Weak and outdated regulation	FDA and FTC not aligned with platform dynamics	Map enforcement gaps	Update regulations; FDA-FTC coordination; international alignment
Low digital literacy limits user judgment	Limited ability to assess online drug info	Test literacy interventions	Education campaigns; target youths and high-risk groups

Abbreviations: FDA, US Food and Drug Administration; FTC, Federal Trade Commission.

### Limitations

This review has several limitations. First, the evidence base for prescription drug promotion by social media influencers remains small and fragmented, reflecting the underresearched nature of this emerging phenomenon. Second, the included studies were heterogeneous in design, methods, and outcomes, limiting comparability and synthesis and highlighting the need for greater conceptual and methodological standardization. Third, most studies relied on qualitative, observational, or controlled experimental designs, restricting causal inference about clinical effects on medication use, prescribing behavior, or health outcomes. Finally, the restriction to English-language, academic literature may have excluded relevant gray literature, regulatory reports, and studies from non–English-language contexts, potentially contributing to an overrepresentation of research focused on platforms commonly used in English-speaking countries.

## Conclusions

This systematic scoping review synthesized the emerging literature on prescription drug promotion by social media influencers and identified recurring concerns related to misinformation, weak and inconsistent regulatory oversight, and the persuasive influence of personal and parasocial narratives. Across study designs and contexts, the evidence suggested that influencer-driven promotion often blurs the boundary between personal experience and commercial messaging, making it difficult for audiences to recognize promotional intent and critically evaluate drug-related claims.

Although the evidence base remains limited and fragmented, the consistency of findings across studies points to several public health risks. Emotionally resonant storytelling, low health literacy, and insufficient disclosure practices may amplify the impact of misleading or incomplete information about prescription drugs, while existing regulatory frameworks and enforcement mechanisms appear ill-equipped to address these dynamics.

Together, these findings highlight the need for updated and enforceable regulatory guidance, standardized disclosure requirements, and stronger platform accountability. Future research should examine clinical behavioral and health outcomes and evaluate regulatory and disclosure interventions to inform evidence-based policy responses. Proactive engagement by researchers, regulators, and platforms is essential to address this evolving form of pharmaceutical promotion and protect public health.

## Notes

Supplement 1. **eTable 1**. Example of Search String Used on APA PsycInfo **eTable 2. **Predeveloped Codebook

Supplement 2.
Data Sharing Statement
