# Supplementary material for: Prescription Drug Promotion by Social Media Influencers: A Systematic Scoping Review
Source: JAMA Netw Open. 2026 Mar 23;9(3):e262738. doi: 10.1001/jamanetworkopen.2026.2738 (PMC13010202; doi:10.1001/jamanetworkopen.2026.2738)
Supplement: Supplement 1. — eTable 1. Example of Search String Used on APA PsycInfo eTable 2. Predeveloped Codebook [file jamanetwopen-e262738-s001.pdf]

Supplementary Online Content

Gell S, Dave S, Willis E, Vitale EJ, Woloshin S, Heiss R. Prescription drug promotion by social media influencers: a systematic scoping review. *JAMA Netw Open.* 2026;9(3):e262738. doi:10.1001/jamanetworkopen.2026.2738

eTable 1. Example of Search String Used on APA PsycInfo

eTable 2. Predeveloped Codebook

This supplementary material has been provided by the authors to give readers additional information about their work.

© 2026 Gell S et al. *JAMA Network Open*.

eTable 1. Example of Search String Used on APA PsycInfo

Concept	Search String
Social Media Influencers	DE "Fame" OR DE "Celebrities" OR DE "Parasocial Interaction" OR TI ((fame or famous or influencer* or microinfluencer* or "blue tick" or bluetick or "blue check" or bluecheck or ambassador* or endorser* or celebrit* or microcelebrit* or parasocial or "para social" or vlogger* or blogger* or instagrammer* or youtuber* or streamer* or entrepreneur* or podcast* or maven* or spokes* or "public figure*") or ((verified or classified or content) W2 creator*)) OR AB ((fame or famous or influencer* or microinfluencer* or "blue tick" or bluetick or "blue check" or bluecheck or ambassador* or endorser* or celebrit* or microcelebrit* or parasocial or "para social" or vlogger* or blogger* or instagrammer* or youtuber* or streamer* or entrepreneur* or podcast* or maven* or spokes* or "public figure*") or ((verified or classified or content) W2 creator*))
Social Media Platforms	DE "Blog" OR DE "Social Media" OR DE "Online Social Networks" OR DE "Social Marketing" OR DE "Online Community" OR DE "Internet" OR TI ((social W1 (application* or apps or media or network* or platform* or website* or communit* or market* or algorithm*)) or (meta or metaverse or instagram* or youtube* or twitter or tweet* or facebook or tiktok or snapchat or vk or weibo or blog* or vlog* or wechat or weixin or qq or qzone or douyin or kuaisho or "baidu tieba" or discord or twitch or linkedin or xing or whatsapp or reddit or signal or telegram or pinterest or myspace or tumbl* or kik or imessag* or sms or vine or forum* or direct messag* or dm* or chat* or feed* or follow* or comment* or podcast* or livestream* or "live stream*" or reels or "online network*") or (dcta or "direct to consumer" or dtc or advert*)) OR AB ((social W1 (application* or apps or media or network* or platform* or website* or communit* or market* or algorithm*)) or (meta or metaverse or instagram* or youtube* or twitter or tweet* or facebook or tiktok or snapchat or vk or weibo or blog* or vlog* or wechat or weixin or qq or qzone or douyin or kuaisho or "baidu tieba" or discord or twitch or linkedin or xing or whatsapp or reddit or signal or telegram or pinterest or myspace or tumbl* or kik or imessag* or sms or vine or forum* or direct messag* or dm* or chat* or feed* or follow* or comment* or podcast* or livestream* or "live stream*" or reels or "online network*") or (dcta or "direct to consumer" or dtc or advert*))
Prescription Drugs	DE "Self-Medication" OR DE "Prescription Drugs" OR DE "Prescribing (Drugs)" OR TI (medicat* or drug or prescri* or pharmaceutical or "off label" or unauthorized or unregulated or unlicensed or "over the counter" or otc or Phrma or Efpia) OR AB (medicat* or drug or prescri* or pharmaceutical or "off label" or unauthorized or unregulated or unlicensed or "over the counter" or otc or Phrma or Efpia)

© 2026 Gell S et al. *JAMA Network Open*.

eTable 2. Predeveloped Codebook

Topic	Category	Description
Metadata	Authors	Authors of the publication (order as indicated in the article).
	Title	Title of publication.
	Year	Year of Publication
	Journal	Journal in which the articles have been published.
	Publisher	Publisher of the Journal.
	Country Location	Continent of the country the data was collected or affiliation of the first author.
	Study Funding	Funding body of the published study.
	Topics	Best fitting topic for the drug related topic of the article.
Study Design	Approach	Indicate if the article is empirical or theoretical.
	Objective - Key Issue	Main objective or main issues addressed by the article.
	Outcome Measure	Type of measurement for main (dependent) variable.
	Design	Method(s) of data collection (if more than one separate by comma).
	Theory	Main theories stated to investigate the research objective.
	Target Group	Addressed target group in the article.
	Sample Size	Sample size of the researched target group in the article.
	Sample Characteristics	Characteristics of the sample included in the study (i.e. Age, Gender).
Influencer Information	Definition of Influencer	Does the article provide a concrete definition of influencers - provide the mentioned definition.
	Size of Influencer Audience	Select the appropriate category of researched influencers.
	Influencer Type	Specific influencer types researched in the article.
	Influencer Demographics	If addressed in the article, provide demographics of the researched influencers (e.g. Age, Gender).
	Sponsored Content	Is the influencer content addressed in the article sponsored?

© 2026 Gell S et al. *JAMA Network Open*.

	Social Media Platform	Which social media platforms are mentioned in the article (background, methods, findings etc.)?
Influencer Content	Post Format	Select the post formats influencers used for prescription drug marketing
	General Content	Select the topics that best suit the general content disseminated by the researched influencers.
	Disease, Condition, Problem	Which issues are addressed by the researched influencer content?
	Disease - Other	If 'other' was selected in the previous column, provide a description here.
Prescription Drug Information	Pharmaceutical Company	Provide the name of the pharmaceutical company distributing the drug.
	Drug Name	Specific drug brands researched in the article.
	Pharmaceutical Substance	Name the active pharmaceutical ingredient in the researched drug(s).
	Promoted Drug Use	Select the promoted use of drugs in researched Influencer content.
Findings	Main Results	Summary of main findings based on results and discussion section.
	Limitations Authors	Limitations discussed by the authors of the article.
	Limitations Team	Limitations of the study by the research team.
Regulation and Research	Policies and ToS	Does the article address policies or terms of services related to prescription drug marketing.
	Policy and ToS Names	Provide the name of the policy or the Platform introducing the ToS.
	Monitoring and Enforcement	Does the article evaluate monitoring and/or enforcement of prescription drug marketing.
	Recommendations for Action and Research	Author recommendations for research or (policy) action.

© 2026 Gell S et al. *JAMA Network Open*.
