# Supplementary material for: Prescription Drug Promotion by Social Media Influencers: A Systematic Scoping Review
Source: JAMA Netw Open. 2026 Mar 23;9(3):e262738. doi: 10.1001/jamanetworkopen.2026.2738 (PMC13010202; doi:10.1001/jamanetworkopen.2026.2738)
Supplement: Supplement 2. — Data Sharing Statement [file jamanetwopen-e262738-s002.pdf]

Data Sharing Statement

Gell. Prescription Drug Promotion by Social Media Influencers. *JAMA Netw Open*. Published March 23, 2026. doi:10.1001/jamanetworkopen.2026.2738

Data
Data available: No

Additional Information
Explanation for why data not available: This study is a systematic scoping review and did not involve the collection of individual patient data. All data analyzed are available in the published articles included in the review and cited in the manuscript.
